# Spatiotemporal analysis of tuberculosis incidence on the border between Brazil and Argentina: a time series study, 2009-2021

**DOI:** 10.1590/S2237-96222025v34e20240650.en

**Published:** 2025-10-20

**Authors:** Juliane Jose Massignani Perotto, Maria de Jesus Mendes da Fonseca, José Ueleres Braga

**Affiliations:** 1 Fundação Oswaldo Cruz, Rio de Janeiro, RJ, Brasil

**Keywords:** Tuberculosis, Incidence, Border Areas, Spatial Analysis, Time Series Studies, Tuberculosis, Incidencia, Áreas Fronterizas, Análisis Espacial, Estudios de Series Temporales

## Abstract

**Objective:**

To analyze tuberculosis cases in the international border region between the state of Santa Catarina, Brazil, and the province of Misiones, Argentina, from 2009 to 2021.

**Methods:**

This was a time series study with geocoding of the average tuberculosis incidence rates of reported tuberculosis cases. Choropleth maps were plotted to identify the spatial distribution pattern and verify changes between the pre-COVID-19 pandemic period (2009 to 2019) and the full study period (2009 to 2021). Global and local Moran indices were used for spatial analyses, and segmented linear regression using the joinpoint regression method was employed for temporal analysis.

**Results:**

We identified a heterogenous tuberculosis spatial distribution pattern, positive spatial autocorrelation in both periods (Moran’s index 0.177 and 0.178; p-value 0.020), presence of spatial clusters and non-significant changes in temporal trends, were not significant, average annual percentage change was 4.0 and the 95% confidence intervals ranged from -1.7 to 10.0. There was no change in case recording during the COVID-19 pandemic. Being adult and of the male sex were predominant characteristics of the patients.

**Conclusion:**

No significant changes in the temporal trend of incidence were detected, but spatial clusters of the high-high type (municipality and neighboring areas with high incidence) were located in Argentina and low-low clusters (municipalities and neighboring areas with low incidence) in Brazil.

## Resumen

**Objetivo:**

Analizar los casos de tuberculosis en el área fronteriza internacional entre el estado de Santa Catarina. Brasil, y la provincia de Misiones, Argentina, entre 2009 y 2021.

**Métodos:**

Estudio de series temporales con geocodificación de las tasas promedio de incidencia de tuberculosis de los casos notificados. Se construyeron mapas coropléticos para identificar el patrón de distribución espacial y verificar la variación entre el período previo a la pandemia de COVID-19 (2009-2019) y el período completo del estudio (2009-2021). Se utilizaron los índices de Moran global y local para los análisis espaciales, y la regresión lineal segmentada mediante el método de regresión de puntos de unión para el análisis temporal.

**Resultados:**

El patrón de distribución espacial de la tuberculosis fue heterogéneo, la autocorrelación espacial fue positiva en ambos períodos (índice de Moran 0,177 y 0,178; valor p 0,020), la presencia de conglomerados espaciales y cambios en las tendencias temporales no fueron significativos, el cambio porcentual anual medio fue de 4,0 y los intervalos de confianza del 95% fueron de -1,7 a 10,0. No hubo variación en el registro de casos durante la pandemia de COVID-19. El grupo de edad adulta y el sexo masculino fueron características predominantes de los pacientes.

**Conclusión:**

No se detectaron cambios significativos en la tendencia temporal de la incidencia, pero se localizaron conglomerados espaciales del tipo alto-alto (municipio y áreas vecinas con alta incidencia) en Argentina y del tipo bajo-bajo (municipios y áreas vecinas con baja incidencia) en Brasil.

**Ethical aspects**

This research used public domain anonymized databases.

## Introduction

Tuberculosis is an infectious disease caused by the *Mycobacterium tuberculosis* bacillus. It is an ancient disease and its incidence increases mainly when the population is subject to poor socioeconomic conditions (1). 

It is estimated that a quarter of the global population is infected with the tuberculosis bacillus. In 2023, 10.8 million new tuberculosis cases were recorded, equivalent to an incidence rate of 134 cases per 100,000 inhabitants. In the Americas, Brazil and Argentina had incidence rates of 49 and 35 cases per 100,000 inhabitants, respectively (1).

Studies on tuberculosis in Latin American country borders are rare. A study conducted in the border region of Colombia with Venezuela analyzed a municipality in the Colombian border area, but did not include municipalities from both countries (2). 

Due to the inherent vulnerabilities of border areas, such as population mobility, inadequate living conditions, and limited access to health services, these areas have a higher risk of tuberculosis transmission. Controlling this disease is even more difficult because it does not depend on just one country, but on all countries that share a border. The epidemiological situation of tuberculosis may be worsened by the low number of patients and case underreporting, which make it difficult to identify areas with high incidence that are priorities for disease control actions (3).

The area covered by this study, which comprises a twin-city conurbation zone, had a high population flow and concentration of vulnerable groups, which made the scenario favorable to occurrence of tuberculosis and made its investigation necessary. In addition, a study on tuberculosis carried out on the Brazil/Paraguay/Argentina triple frontier identified an increasing trend in tuberculosis transmission in this area in the period 2001-2007 (4). The epidemiological situation of tuberculosis in the world, the diagnosis and reporting of which has reduced, worsened with the COVID-19 pandemic (5), declared by the World Health Organization (WHO) between March 2020 and May 2023.

In view of this, despite the incidence rates of tuberculosis being known in most states and provinces of Brazil and Argentina, few studies have performed a temporal and spatial series analysis of tuberculosis incidence in this border area. This knowledge is important for supporting transnational public policies, aligned with global goals such as the WHO’s “End Tuberculosis Strategy”.

This study aimed to analyze tuberculosis cases in the international border region between the state of Santa Catarina, Brazil, and the province of Misiones, Argentina, between 2009 and 2021.

## Methods

### Study design 

This was a time series study of tuberculosis incidence, taking as its units of analysis the municipalities located in the international border region between the state of Santa Catarina, Brazil, and the province of Misiones, Argentina, between 2009 and 2021.

### Study area and population

**Figure 1:**
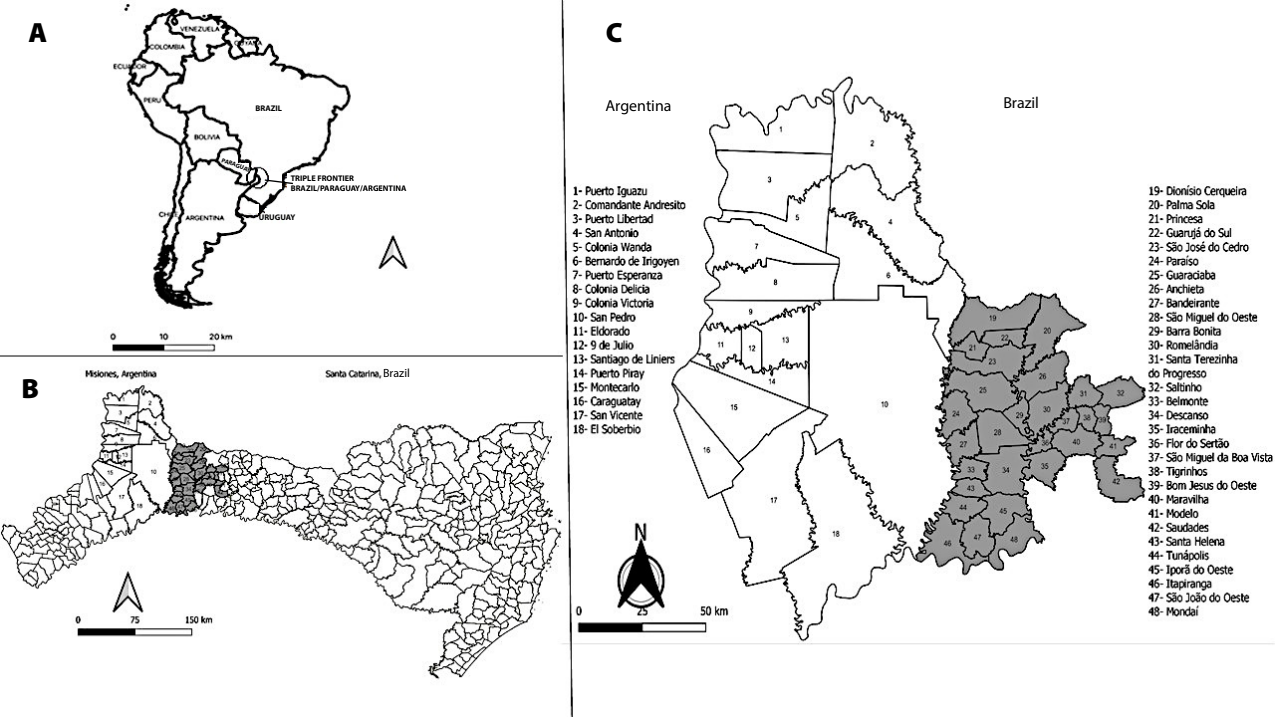
International border between Santa Catarina and Misiones: (A) location of the countries in South America; (B) location of Misiones, Argentina, and Santa Catarina, Brazil; (C) location of the study region

Santa Catarina has 295 municipalities and is located in the Southern region of Brazil. It is part of the Southern Arc of the Brazilian border strip, having 82 municipalities in the border strip with Argentina. Thirty of these are located within Santa Catarina’s far west health region, which is closest to the border, where the study was conducted, with an estimated population of 232,039 inhabitants (6) (Figure 1). 

Misiones is an Argentine province located in the Northeast region of that country and borders Paraguay to the west; Brazil to the east, north and south; and the Argentine province of Corrientes to the southwest. Misiones is divided into 17 departments with 75 municipalities. This study analyzed six departments comprising 18 municipalities in the region closest to the international border with Santa Catarina, the population of which was estimated at 339,043 inhabitants in 2010 (7) (Figure 1).

### Data source

We used secondary data from the tuberculosis case reporting systems of both countries for the period 2009-2021. In Brazil this is the Notifiable Health Conditions Information System (8), while in Argentina it is the National Health Surveillance System (9).

Population data were obtained from the 2010 national censuses, made available in Brazil by the Brazilian Institute of Geography and Statistics (6) and in Argentina by the National Institute of Statistics and Censuses (7).

### Study variable characterization 

The study variables were characterized as dependent and independent. The dependent variable was: incidence (all forms of tuberculosis), defined in Brazil as “new case”, “unknown” and “post-mortem” and in Argentina as “incident” (new cases, recurrent cases or no information on the type of patient). 

The independent variables were: age (age range in years: 0-14, 15-29, 30-44, 45-59, 60-74, 75 or over), sex (female, male), place of residence (reporting municipality in Brazil and municipality of residence in Argentina) and nationality (Brazilian, Argentine).

### Data analysis

The patients’ profiles were characterized by absolute and relative frequencies of their age and sex variables. Comparison of association differences between the two countries was assessed using the chi-square test.

Average incidence rates of reported tuberculosis cases in the general population of each municipality were calculated per 100,000 inhabitants per year and in the pre-COVID-19 pandemic period (2009-2019) and the full study period (2009-2021), which included the initial years of the pandemic, to detect changes between the periods. In addition to the crude rates, age-standardized incidence rates were estimated using the direct method and smoothed rates (global empirical Bayesian and spatial moving average). The spatial analyses performed were: global empirical Bayesian rate, spatial moving average, global and local Moran index (I), and choropleth maps.

Smoothed rates were used in order to detect more accurately spatial distribution patterns of the disease and identify areas of higher incidence. This was justified by the existence of random fluctuation of data in the study area, and the empirical Bayesian estimator method was applied because it is recommended for smoothing crude rates. Smoothed rates were based on the use of information from other areas that make up the study region, reducing instability not associated with the occurrence of the event (10).

In the analyses of the spatial moving average and the Moran index, a queen-type neighboring area matrix was built using as a criterion the existence of a common border between municipalities considering the first-order level (11).

Choropleth maps of the age-adjusted rates and smoothed rates were plotted to identify the spatial distribution pattern and detect changes between the periods.

Existence of spatial autocorrelation of tuberculosis incidence was checked by calculating the global Moran index, which characterizes spatial dependence. The index showed how values were correlated in space. A value greater than zero indicated positive spatial dependence, where neighboring areas tended to have similar incidence rates (11).

The local Moran index was used to locate spatial clusters, as it enabled identification of spatially autocorrelated municipalities that had the most influence. This measure was used due to the use of the Moran index, which is the most commonly used measure of global spatial autocorrelation, and this is its local version. Cluster location could be seen on the local Moran index maps: spatial clusters (+) of the high-high type (municipality and neighboring areas with high incidence) and low-low (municipalities and neighboring areas with low incidence) and spatial atypical locations (-), high-low (municipalities with high incidence and neighboring areas with low incidence) and low-high (municipalities with low incidence and neighboring areas with high incidence). Non-significant locations were not highlighted on the maps (12).

Temporal analysis was performed using the joinpoint regression method, in which annual percentage change (APC) for each segment and average annual percentage change (AAPC) for the entire period of tuberculosis incidence in the Brazilian and Argentine municipalities between 2009 and 2021 were estimated. This method performed segmented linear regression that enabled identification of the indicator’s trend (whether stationary, increasing or decreasing) and the points at which there was a change in this trend, estimating APC and AAPC. The average annual age-adjusted tuberculosis incidence rate was used as the dependent variable (logarithmic transformation of the rate), while the year was taken as the independent variable.

The joinpoint model was adjusted by providing the minimum (zero) and maximum (two) number of points, due to the number of years analyzed, to test whether an apparent change in the trend was significant. Significance tests used the Monte Carlo permutation method. In this case, the null hypothesis is APC or AAPC equal to zero, that is, rates neither increasing nor decreasing. For each trend detected, 95% confidence intervals (95%CI) were considered (13). We used a 5% significance level for all statistical tests performed in this study.

The following software programs were used for data analysis,: R version 4.3 (age-adjusted rates and chi-square test), GeoDa version 1.22 (smoothed rates and global and local Moran indices), QGIS version 3.28.11 (choropleth maps) and Joinpoint Regression Program version 5.0 (temporal analysis).

## Results

In the period 2009-2021, 1,040 cases of any form of tuberculosis were reported in the 48 municipalities within the study area, with 637 cases (61.2%) in male patients and 393 cases (37.8%) in female patients. The largest number of cases was distributed between the 45-59 age group in Brazil and the 15-29 age group in Argentina, followed by the 30-44 age group in both countries. The statistical difference obtained via the chi-square test for age group and sex in both countries was significant (Table 1).

**Figure 2:**
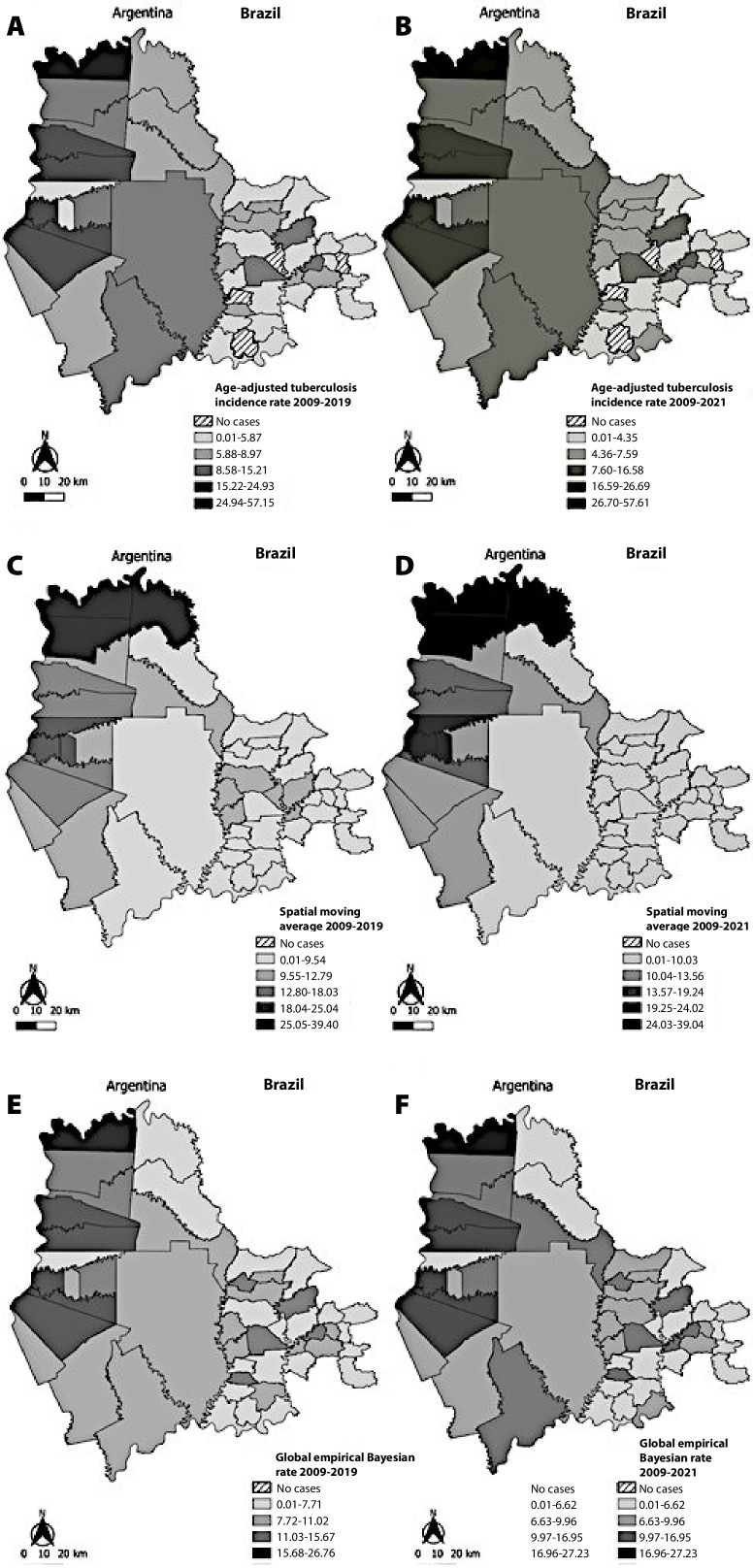
Average tuberculosis incidence rates per 100,000 inhabitants in Brazilian and Argentine municipalities: (A) age-adjusted rate, 2009-2019, (B) age-adjusted rate, 2009-2021, (C) spatial moving average, 2009-2019, (D) spatial moving average, 2009-2021, (E) global empirical Bayesian rate, 2009-2019, (F) global empirical Bayesian rate, 2009-2021

Geocoding of age-adjusted tuberculosis incidence rates in the two periods showed heterogeneous distribution. There were municipalities with rates above 50 cases per 100,000 inhabitants in the northern part of the study area, municipalities in the eastern part with rates below five cases per 100,000 inhabitants, and municipalities in the southern and central-eastern parts with intermediate incidence rates, not exceeding 17 cases per 100,000 inhabitants (Figure 2).

A heterogeneous spatial distribution pattern of the disease was observed in the maps of the spatial moving average of tuberculosis incidence in both periods, with municipalities presenting high rates in the northern area and municipalities with lower rates in the eastern area (Figure 2).

The spatial distribution pattern indicated by the global empirical Bayesian smoothed tuberculosis incidence rates was also heterogeneous in both periods. In the map of the full period, municipalities with high rates were identified in the northern (Puerto Iguazu-Misiones), northwest (Puerto Esperanza and Colonia Delícia-Misiones) and southwest (Eldorado, Puerto Piray and Montecarlo-Misiones) parts of the study area, while municipalities with lower rates were identified in the eastern part. In the pre-pandemic period, a more homogeneous distribution was observed in the southern and central parts of the study area (Figure 2).

**Figure 3:**
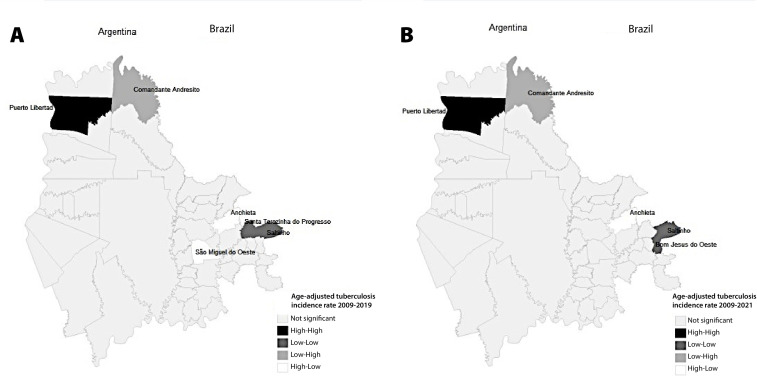
Location of spatial clusters, according to the local Moran index in Brazilian and Argentine municipalities: (A) period 2009-2019 and (B) period 2009-2021

Moran’s global index revealed the existence of positive spatial autocorrelation of the disease in the study area, with I = 0.177 (p-value 0.020) in the pre-pandemic period and I = 0.178 (p-value 0.020) in the full period. Moran’s local index confirmed the existence of spatial dependence in both study periods and indicated the location of spatial clusters and atypical locations. In the full period, spatial clusters were identified - one high-high municipality (Puerto Libertad-Misiones) and two low-low municipalities (Saltinho and Bom Jesus do Oeste-Santa Catarina) - and spatial atypical locations - one low-high municipality (Comandante Andresito-Misiones) and one high-low municipality (Anchieta-Santa Catarina). This was similar in the pre-pandemic period, but included one more high-low municipality (São Miguel do Oeste-Santa Catarina) and the change of a low-low municipality (Santa Terezinha do Progresso, replacing Bom Jesus do Oeste-Santa Catarina) (Figure 3).

Temporal analysis using the joinpoint regression method suggested three temporal trends for the period 2009-2021, which were similar for Brazilian municipalities, Argentine municipalities and municipalities of both countries, but they were not statistically significant. The first segment suggested an increasing trend for Santa Catarina between 2009 and 2016 (APC 9.3; 95%CI -36.8; 107.8) and for Misiones between 2009 and 2012 (APC 14.7; 95%CI -5.3; 55.2). The AAPC also suggested an increasing trend for both, being greater in the Argentine part (AAPC 4.1; 95%CI -0.9; 9.1) (Table 2).

## Discussion

The results revealed a heterogeneous spatial distribution pattern of tuberculosis incidence, positive spatial autocorrelation, and the presence of high-high spatial clusters in Argentina and low-low clusters in Brazil, but no significant temporal trend changes were identified in the study area. Furthermore, the results demonstrated that there was no change in the reporting of tuberculosis cases during the COVID-19 pandemic and that the adult age group and male sex were the predominant characteristics of the patients.

This study had limitations related to the use of secondary databases, the inherent characteristics of each country’s surveillance system and the differences between the divisions of the municipalities of the two countries. However, through the analytical techniques used, it was possible to overcome some of these limitations and identify the areas of high risk for tuberculosis transmission.

The patient profile identified corroborates those found in studies where the economically active population, the adult age group and males were the most affected by tuberculosis (14-16). This profile observed in the border region is similar to that of the two countries studied, where, in Brazil, of the 59,735 new cases of pulmonary tuberculosis reported in 2021, 70.1% occurred in males, in the 20-34 age group, followed by the 50-64 age group (5), and in Argentina, in 2020, 56.7% of reported cases were males and 61.8% occurred in the 15-44 age group (17).

The heterogeneous spatial distribution pattern of tuberculosis incidence, i.e., non-uniform, presented in the study area was in accordance with other national (4,18-19) and international studies that also identified this distribution pattern for cases of the disease (2,20).

The existence of positive spatial autocorrelation of tuberculosis indicated that neighboring municipalities have similar rates (high or low). This suggests the probable presence of analogous risk factors in these areas (environmental, social, economic, etc.), which may explain the similarity observed (21).

The municipalities identified as high-high spatial clusters were located in the Argentine part, indicating greater transmission of *Mycobacterium tuberculosis* and greater risk for the population. The low-low spatial clusters were located in the Brazilian part, where the lowest incidence rates of the disease were recorded, which draws attention, among other things, to case underreporting (21). The municipalities identified as spatial atypical locations deserve attention, due to the high rates of their neighboring municipalities, as they run the risk of also becoming high.

In the northern part of the study area, located in the Brazil/Paraguay/Argentina triple frontier region, the municipality of Puerto Iguazu-Misiones had the highest tuberculosis incidence in the period (57.6 cases per 100,000 inhabitants), higher than the national Argentine average (22.6 cases per 100,000 inhabitants) and higher than the overall rate of the province of Misiones (19 cases per 100,000 inhabitants) in 2020 (17). Research on tuberculosis carried out in the triple frontier region, covering the period 2001-2007, identified an increasing trend in the transmission of the disease in this region and in the municipality of Puerto Iguazu-Misiones. In addition, it identified that the places with the highest incidence were in an area where the municipalities also border Paraguay, which demonstrated the influence of this region on *Mycobacterium tuberculosis* transmission (4). 

High incidence of tuberculosis in Argentina may be due to high population density, social inequalities found in large urban centers (22) and the fact that the northern area is within the triple frontier region (4). This pattern of disease corroborates the study carried out in Northeast Brazil that also identified high tuberculosis incidence rates in large cities and their metropolitan regions (23).

International border regions have shown higher tuberculosis incidence than other spatial areas having, among the main factors associated with disease transmission: high population mobility, migratory movements, activities with the following main associated factors: disease transmission, high population mobility, migratory movements, activities with high environmental impact, lack of or difficulty in accessing health services, and the population’s living conditions (4, 24). The data found on tuberculosis incidence in this study did not allow us to conclude that there is a risk of becoming ill from tuberculosis in this border area, although they indicated indicate greater influence on tuberculosis transmission in the triple frontier region.

The lowest tuberculosis incidence rates disease were recorded in the eastern part of the study area, where the Brazilian municipalities are located, and some municipalities were identified as not having any reported cases during the entire study period. This data highlights the quality of health services in this area with regard to tuberculosis. In Brazil, the highest incidence of infectious and parasitic diseases, including tuberculosis, is found in the country’s North and Midwest regions and the mid-north subregion of the Northeast region, decreasing in the Southern region, where Santa Catarina is located, and on the eastern coast. This decrease is associated, among other premises, with better population living conditions (25). The municipal human development index of the Brazilian municipalities in this study ranged from medium to very high, compatible with good population living conditions (6).

These discrepant differences in tuberculosis incidence between Brazilian and Argentine municipalities also indicated a change in the behavior of the disease in the population, which can be attributed to factors such as the influence and living conditions of the population and the quality of surveillance, diagnosis and treatment, as they influence tuberculosis control (25,26). Case underreporting can also be suspected in these areas of lower incidence, since they are located close to areas with high tuberculosis transmission and where the flow of people between the two countries is high. It is recognized that, with regard to the dynamics of tuberculosis transmission, population interactions are important and decisive in tuberculosis bacillus infection (21).

The analysis of the different study periods, i.e. the pre-pandemic period (2009-2019) and the full period (2009-2021), did not reveal major changes in tuberculosis incidence. This area did not follow the trend of several places around the world that, in general, reported a decrease in tuberculosis case reporting during the COVID-19 pandemic, with underreporting and difficulty in accessing health services being possible causes (27). In Argentina, the tuberculosis notification rate per 100,000 inhabitants in the period 2014-2019 increased, with an average annual change of 3.7%, while in the period 2019-2020, the rate decreased by 12.8%, this also being attributed, among other things, to the COVID-19 pandemic (17). In the neighboring Brazilian state of Paraná, a decrease was observed during the pandemic period, differing from the findings of this study, which did not find this trend. The hypothesis is that tuberculosis notifications in this area showed an increasing trend even before the start of the COVID-19 pandemic (28).

The temporal analysis suggested upward trends in tuberculosis incidence in the study region, but these trends were not significant. However, data has shown an increase in Brazil from 2015 onwards, where tuberculosis incidence was 34.3 cases per 100,000 inhabitants, increasing to 36.9 in 2018, stabilizing in 2019 (37.1 cases per 100,000 inhabitants) (5). In Argentina, the rate also increased from 22.8 cases per 100,000 inhabitants in 2015 to 25.5 in 2021 (17). This trend was heterogeneous across the states and provinces of both countries and showed increasing and decreasing annual changes (5,17).

These increases in tuberculosis incidence may be related to social issues, diagnostic problems, treatment dropout, tuberculosis-HIV co-infection (29) and drug-resistant tuberculosis. Incorporation of new, more sensitive diagnostic technologies into the Brazilian National Health System may contribute to the increase in incidence detection (5,30). Argentina has also made progress in tuberculosis diagnosis with the introduction of new technologies and health professional training (18). These data indicate that tuberculosis remains a challenging public health problem and hinders the achievement of the WHO “End Tuberculosis Strategy” target of reducing tuberculosis incidence to less than 10 cases per 100,000 population by 2035 (30). 

By analyzing international border municipalities as a single area, it was possible to identify the areas with the highest risk of tuberculosis transmission, which are a priority for disease control actions, and those with the lowest incidence, where it is proposed to investigate the epidemiological scenario of tuberculosis and surveillance actions in more detail.

No significant changes in the temporal trend of tuberculosis incidence were detected, but spatial clusters of the high-high type (municipality and neighboring areas with high incidence) were located in Argentina and low-low (municipalities and neighboring areas with low incidence) in Brazil. These results suggested integrated actions by the two countries, such as diagnostic campaigns in areas of higher risk, and actions to improve surveillance in areas of underreporting. In addition, they highlight the need for transnational cooperation to strengthen tuberculosis control in border areas. This includes improving public policies, bilateral actions for health service funding and care, shared information system management, and standardized surveillance services.
