# Supplementary material for: Spatiotemporal analysis of tuberculosis incidence on the border between Brazil and Argentina: a time series study, 2009-2021
Source: Epidemiol Serv Saude. 2025 Oct 20;34:e20240650. doi: 10.1590/S2237-96222025v34e20240650.en (PMC12536964; doi:10.1590/S2237-96222025v34e20240650.en)
Supplement: Translation

# Translation: Análise espaço-temporal da incidência de tuberculose na fronteira entre Brasil e Argentina: estudo de série temporal, 2009-2021

**DOI:** 10.1590/S2237-96222025v34e20240650.pt

**Authors:** Juliane Jose Massignani Perotto, Maria de Jesus Mendes da Fonseca, José Ueleres Braga

**Affiliations:** 1 Fundação Oswaldo Cruz, Rio de Janeiro, RJ, Brasil

**Keywords:** Tuberculose, Incidência, Áreas de Fronteira, Análise Espacial, Estudos de Séries Temporais

## Abstract

**Objetivo:**

Analisar os casos de tuberculose na região de fronteira internacional entre Santa Catarina e a província de Misiones, Argentina, no período 2009-2021.

**Métodos:**

Tratou-se de estudo de série temporal com geocodificação das taxas médias de incidência de tuberculose dos casos notificados de tuberculose. Foram construídos mapas coropléticos para identificar o padrão de distribuição espacial e verificar a variação entre o período pré-pandêmico da covid-19 (2009-2019) e período completo do estudo (2009-2021). Utilizaram-se os índices de Moran global e local para as análises espaciais e, para a análise temporal, a regressão linear segmentada pelo método de regressão do joinpoint.

**Resultados:**

Identificou-se que o padrão de distribuição espacial da tuberculose era heterogêneo, a autocorrelação espacial foi positiva em ambos os períodos (índice de Moran 0,177 e 0,178; p-valor 0,020), a presença de aglomerados espaciais e mudanças de tendência temporal não significativas, a variação percentual anual média foi 4,0 e os intervalos de confiança de 95% foram -1,7 a 10,0. Não houve variação no registro de casos durante a pandemia da covid-19. A faixa etária adulta e o sexo masculino foram características predominantes dos pacientes.

**Conclusão:**

Não foram detectadas mudanças de tendência temporal significativas da incidência, mas foram localizados aglomerados espaciais dos tipos alto-alto (município e vizinhos com alta incidência) na Argentina e baixo-baixo (municípios e vizinhos com baixa incidência) no Brasil.

**Aspectos éticos**

Esta pesquisa utilizou bancos de dados de domínio público e anonimizados.

## Introdução

A tuberculose é uma doença infectocontagiosa causada pelo bacilo *Mycobacterium tuberculosis*. A incidência da tuberculose, uma doença milenar, tem seu risco aumentado sobretudo por condições socioeconômicas deficitárias da população (1). 

Estima-se que um quarto da população mundial se encontra infectada com o bacilo da tuberculose. Em 2023, foram registrados 10,8 milhões de casos novos de tuberculose, equivalente à incidência de 134 casos por 100 mil habitantes. Na região das Américas, o Brasil e a Argentina obtiveram incidência de 49 e 35 casos por casos por 100 mil habitantes (1).

Estudos sobre a tuberculose nas fronteiras da América Latina são raros. Um estudo realizado na região de fronteira da Colômbia analisou um município da área de fronteira, mas não incluiu municípios dos dois países (2). 

Por causa das vulnerabilidades intrínsecas às áreas de fronteira, como mobilidade populacional, condições de vida inadequadas e acesso limitado aos serviços de saúde, essas áreas apresentaram maior risco de transmissão da tuberculose. O controle dessa doença é ainda mais difícil, porque não depende somente de um país, mas de todos que têm fronteira comum. A tuberculose pode ter sua situação epidemiológica agravada devido à baixa captação de doentes e à subnotificação de casos que dificultam o reconhecimento de áreas de elevada incidência que são prioritárias para as ações de controle da doença (3). 

A área deste estudo, que compreende uma zona de conurbação de cidades gêmeas, apresentou elevado fluxo populacional e concentração de grupos vulneráveis, o que tornou o cenário favorável à ocorrência da tuberculose e sua investigação necessária. Além disso, uma pesquisa sobre a tuberculose realizada na tríplice fronteira Brasil/Paraguai/Argentina identificou tendência de aumento na transmissão da doença nessa região no período 2001-2007 (4). A situação epidemiológica da tuberculose no mundo, com redução no diagnóstico e na notificação, se agravou com a pandemia da covid-19 (5), decretada pela Organização Mundial da Saúde (OMS) entre março de 2020 e maio de 2023.

Diante disso, apesar de se conhecer as incidências na maioria dos estados do Brasil e da Argentina, poucos estudos fizeram uma análise de série temporal e espacial da incidência de tuberculose nessa área de fronteira. Esse conhecimento é importante para subsidiar políticas públicas transnacionais, alinhadas às metas globais como a “Estratégia pelo Fim da Tuberculose” da OMS. 

Este estudo teve como objetivo analisar os casos de tuberculose na região de fronteira internacional entre Santa Catarina e a província de Misiones, Argentina, no período 2009-2021.

## Métodos

### Delineamento do estudo

Tratou-se de estudo de série temporal da incidência de tuberculose, tendo como unidades de análise os municípios da região de fronteira internacional entre Santa Catarina e a província de Misiones, Argentina, no período 2009-2021.

### Área e população do estudo

Santa Catarina possui 295 municípios e localiza-se na região Sul do Brasil. Pertence ao Arco Sul da faixa de fronteira brasileira, contemplando 82 municípios na faixa de fronteira com a Argentina. Trinta destes pertencem à região de saúde do extremo oeste de Santa Catarina, região mais próxima da fronteira, onde foi realizado o estudo, com população estimada de 232.039 habitantes (6) (Figura 1). 

**Figura 1:** Fronteira internacional entre Santa Catarina e Misiones: (A) localização dos países na América do Sul; (B) localização de Misiones, Argentina, e Santa Catarina, Brasil; (C) localização da região de estudo

Misiones é uma província da Argentina situada ao nordeste do país e faz fronteira ao Oeste com o Paraguai; ao Leste, Norte e Sul com o Brasil; e ao Sudoeste com a província de Corrientes. Misiones é dividida em 17 departamentos com 75 municípios. Foram analisados neste estudo seis departamentos com 18 municípios na região mais próxima à fronteira internacional com Santa Catarina cuja população foi estimada em 339.043 habitantes (7) (Figura 1). 

### Fonte de dados

Utilizaram-se dados secundários dos sistemas de notificação de casos de tuberculose dos dois países no período 2009-2021, sendo no Brasil o Sistema de Informação de Agravos de Notificação (8) e na Argentina o Sistema Nacional de Vigilância Sanitária (9). 

Os dados populacionais foram obtidos dos censos nacionais de 2010, disponibilizados no Brasil pelo Instituto Brasileiro de Geografia e Estatística (6) e na Argentina pelo Instituto Nacional de Estatística e Censos (7).

### Caracterização das variáveis do estudo

As variáveis do estudo foram caracterizadas como dependente e independentes. A variável dependente foi: incidência (tuberculose todas as formas), definida no Brasil como “caso novo”, “não sabe” e “pós-óbito” e na Argentina como “incidentes” (casos novos, recidivas ou sem informação sobre o tipo de paciente). 

As variáveis independentes foram: idade (faixa etária em anos: 0-14, 15-29, 30-44, 45-59, 60-74, 75 ou mais), sexo (feminino, masculino), local de residência (município de notificação no Brasil e município de residência na Argentina) e nacionalidade (brasileiro, argentino).

### Análise de dados

O perfil dos pacientes foi caracterizado por frequências absolutas e relativas das variáveis idade e sexo. A comparação da diferença de associação entre os dois países foi avaliada por teste qui-quadrado.

Foram calculadas taxas médias de incidência dos casos notificados de tuberculose da população geral de cada município por 100 mil habitantes por ano e nos períodos pré-pandêmico da covid-19 (2009-2019) e período completo do estudo (2009-2021), que incluiu os anos iniciais da pandemia, para detectar variação entre os períodos. Além das taxas brutas, foram estimadas as taxas de incidência padronizadas por idade pelo método direto e as taxas suavizadas (bayesiana empírica global e média móvel espacial). As análises espaciais realizadas foram: taxa bayesiana empírica global, média móvel espacial, índice de Moran global (I) e local e mapas coropléticos. 

As taxas suavizadas foram empregadas com o objetivo de detectar, de modo mais acurado, os padrões de distribuição espacial da doença e identificar zonas de maior incidência. Isso se justificou pela existência de flutuação aleatória dos dados na área de estudo, e o método do estimador bayesiano empírico foi aplicado por ser recomendado para a suavização das taxas brutas. As taxas suavizadas basearam-se no uso de informação das outras áreas que compõem a região de estudo, diminuindo a instabilidade não associada à ocorrência do evento (10).

Nas análises da média móvel espacial e do índice de Moran, foi construída uma matriz de vizinhança do tipo rainha tendo como critério a existência de fronteira comum entre os municípios considerando o nível de primeira ordem (11).

Mapas coropléticos das taxas ajustadas por idade e das taxas suavizadas foram construídos para a identificação do padrão de distribuição espacial e a detecção de variação entre os períodos.

A existência de autocorrelação espacial da incidência de tuberculose foi verificada pelo cálculo do índice de Moran global que caracteriza a dependência espacial. O índice mostrou como os valores estavam correlacionados no espaço. Um valor maior do que zero indicava dependência espacial positiva onde as áreas próximas tendiam a apresentar taxas de incidência semelhantes (11).

O índice de Moran local foi utilizado para localizar os aglomerados espaciais, pois permitiu identificar os municípios autocorrelacionados espacialmente e que mais influenciavam. Essa medida foi utilizada devido ao emprego do índice de Moran que é a medida de autocorrelação espacial global mais comumente utilizada, e essa é a sua versão local. A localização dos aglomerados foi observada nos mapas do índice de Moran local: aglomerados espaciais (+) do tipo alto-alto (município e vizinhos com alta incidência) e baixo-baixo (municípios e vizinhos com baixa incidência) e localidades atípicas espaciais (-), alto-baixo (municípios com alta incidência e vizinhos com baixa incidência) e baixo-alto (municípios com baixa incidência e vizinhos com alta incidência). Localidades não significantes não foram destacadas (12).

A análise temporal foi realizada pelo método de regressão do joinpoint, em que foram estimadas a variação percentual anual (VPA), para cada segmento, e a variação percentual anual média (VPAM), para o período completo, da incidência de tuberculose dos municípios brasileiros, argentinos e ambos entre 2009 e 2021. Esse método executou a regressão linear segmentada que permitiu identificar a tendência do indicador (se estacionária, crescente ou decrescente) e os pontos em que houve modificação dessa tendência estimando a VPA e a VPAM. A taxa anual média de incidência ajustada por idade de tuberculose foi utilizada como variável dependente (transformação logarítmica da taxa), e o ano, como variável independente. 

O modelo de pontos de junção foi ajustado sendo fornecido o número mínimo (zero) e máximo (dois) de pontos, devido ao número de anos analisados, para testar se uma mudança aparente na tendência foi significativa. Os testes de significância utilizaram o método de permutação de Monte Carlo. Neste caso, a hipótese nula é VPA ou VPAM igual a zero, ou seja, as taxas não estavam nem aumentando, nem diminuindo. Para cada tendência detectada, foram considerados intervalos de confiança de 95% (IC95%) (13). O nível de significância de 5% foi considerado para todos os testes estatísticos realizados neste estudo.

Para a análise dos dados, foram utilizados os softwares R versão 4.3 (taxas ajustadas por idade e teste qui-quadrado), GeoDa versão 1.22 (taxas suavizadas e índices de Moran global e local), QGIS versão 3.28.11 (mapas coropléticos) e Joinpoint Regression Program versão 5.0 (análise temporal).

## Resultados

No período 2009-2021, foram notificados 1.040 casos de tuberculose todas as formas nos 48 municípios da área de estudo, sendo 637 casos (61,2%) em pacientes do sexo masculino e 393 casos (37,8%) do sexo feminino. O maior número de casos distribuídos foi entre as faixas etárias 45-59 anos no Brasil e 15-29 anos na Argentina, seguidos de 30-44 anos nos dois países. A diferença estatística obtida pelo teste qui-quadrado para a faixa etária e o sexo nos dois países foi significativa (Tabela 1). 

A geocodificação das taxas ajustadas por idade da incidência de tuberculose nos dois períodos apresentaram distribuição heterogênea. Havia municípios com taxas acima de 50 casos por 100 mil habitantes na zona norte da área de estudo, municípios da zona leste apresentando valores abaixo de cinco casos por 100 mil habitantes e municípios da zona sul e centro-leste com incidências intermediárias, não ultrapassando 17 casos por 100 mil habitantes (Figura 2).

**Figura 2:** Taxas médias de incidência de tuberculose por 100 mil habitantes nos municípios brasileiros e argentinos: (A) taxa ajustada por idade, 2009-2019, (B) taxa ajustada por idade, 2009-2021, (C) média móvel espacial, 2009-2019, (D) média móvel espacial, 2009-2021, (E) taxa bayesiana empírica global, 2009-2019, (F) taxa bayesiana empírica global, 2009-2021

Nos mapas da média móvel espacial da incidência de tuberculose nos dois períodos, observou-se o padrão de distribuição espacial heterogêneo da doença, com municípios apresentando elevadas taxas na zona norte e municípios com menores taxas na zona leste (Figura 2).

O padrão distribuição espacial indicado pelas taxas suavizadas bayesiana empírica global da incidência de tuberculose também foi heterogêneo em ambos os períodos. No mapa do período completo, foram identificados municípios com elevadas taxas nas zonas norte (Puerto Iguazu-Misiones), noroeste (Puerto Esperanza e Colonia Delícia-Misiones) e sudoeste (Eldorado, Puerto Piray e Montecarlo-Misiones) da área de estudo e municípios com menores taxas na zona leste. No período pré-pandêmico, observou-se a distribuição mais homogênea nas zonas sul e central da área de estudo (Figura 2).

O índice de Moran global revelou a existência de autocorrelação espacial positiva da doença na área de estudo, sendo I=0,177 (p-valor 0,020) no período pré-pandêmico e I=0,178 (p-valor 0,020) no período completo. O índice de Moran local confirmou a existência de dependência espacial em ambos os períodos do estudo e indicou a localização dos aglomerados espaciais e das localidades atípicas. No período completo, foram identificados aglomerados espaciais – um município alto-alto (Puerto Libertad-Misiones) e dois municípios baixo-baixo (Saltinho e Bom Jesus do Oeste-Santa Catarina) – e localidades atípicas espaciais – um município baixo-alto (Comandante Andresito-Misiones) e um município alto-baixo (Anchieta-Santa Catarina). No período pré-pandêmico, foi semelhante, porém com a inclusão de mais um município alto-baixo (São Miguel do Oeste-Santa Catarina) e a alteração de um município baixo-baixo (Santa Terezinha do Progresso no lugar de Bom Jesus do Oeste-Santa Catarina) (Figura 3).

**Figura 3:** Localização dos aglomerados espaciais, segundo índice de Moran local nos municípios brasileiros e argentinos: (A) período 2009-2019 e (B) período 2009-2021

A análise temporal pelo método de regressão do joinpoint sugeriu três tendências temporais para o período 2009-2021, sendo estas semelhantes para os municípios brasileiros, argentinos e ambos, entretanto não foram estatisticamente significativas. O primeiro segmento sugeriu tendência crescente para Santa Catarina entre 2009 e 2016 (VPA 9,3; IC95% -36,8; 107,8) e para Misiones entre 2009 e 2012 (VPA 14,7; IC95% -5,3; 55,2). A VPAM também sugeriu tendência de crescimento para ambos, sendo maior na parte Argentina (VPAM 4,1; IC95% -0,9; 9,1) (Tabela 2).

## Discussão

Os resultados revelaram o padrão de distribuição espacial heterogêneo da incidência de tuberculose, autocorrelação espacial positiva, presença de aglomerados espaciais do tipo alto-alto na Argentina e baixo-baixo no Brasil, mas não foram identificadas mudanças de tendência temporal significativas na área de estudo. Além disso, demonstraram que não houve variação no registro de casos de tuberculose durante a pandemia da covid-19 e que a faixa etária adulta e o sexo masculino foram características predominantes dos pacientes. 

Este estudo apresentou limitações no que concerne à utilização de bases de dados secundários, às características inerentes de cada vigilância e às diferenças entre as divisões dos municípios dos dois países. Entretanto, por meio das técnicas analíticas utilizadas, foi possível contornar algumas dessas limitações e identificar as áreas de risco elevado para transmissão da tuberculose.

O perfil identificado dos pacientes corrobora os encontrados em estudos onde a população economicamente ativa, na faixa etária adulta e do sexo masculino foi a mais afetada pela tuberculose (14-16). Esse perfil observado na região de fronteira é semelhante ao dos dois países estudados, onde, no Brasil, dos 59.735 casos novos de tuberculose pulmonar notificados em 2021, 70,1% ocorreram em pessoas do sexo masculino, na faixa etária 20-34 anos seguidos do grupo de 50-64 anos (5), e na Argentina, em 2020, 56,7% dos casos notificados pertenciam ao sexo masculino e 61,8% ocorreram na faixa etária 15-44 anos (17).

O padrão de distribuição espacial heterogêneo da incidência de tuberculose, ou seja, não uniforme, apresentado na área de estudo esteve em conformidade com outras pesquisas nacionais (4,18-19) e internacionais que também identificaram esse padrão de distribuição para os casos da doença (2,20). 

A existência de autocorrelação espacial positiva da tuberculose indicou que os municípios próximos possuem taxas semelhantes (altas ou baixas). Isso sugere a provável presença de fatores de risco análogos nessas áreas (ambiental, social, econômico etc.), que pode explicar a similaridade observada (21).

Os municípios identificados como aglomerados espaciais do tipo alto-alto localizaram-se na parte Argentina indicando maior transmissão do *Mycobacterium tuberculosis* e maior risco para a população. Os aglomerados espaciais do tipo baixo-baixo localizaram-se na parte brasileira onde foram registradas as menores taxas de incidência da doença, o que chama atenção, entre outros, para a subnotificação de casos (21). Os municípios identificados como localidades atípicas espaciais merecem atenção, devido às altas taxas dos seus municípios vizinhos, pois correm o risco de também se tornarem altos. 

Na zona norte da área de estudo, situada em região de tríplice fronteira Brasil/Paraguai/Argentina, o município de Puerto Iguazu-Misiones foi o que apresentou maior incidência de tuberculose no período (57,6 casos por 100 mil habitantes), superior à média nacional do país (22,6 casos por 100 mil habitantes) e da província de Misiones (19 casos por 100 mil habitantes) em 2020 (17). A pesquisa sobre tuberculose realizada na tríplice fronteira, no período 2001-2007, identificou tendência de aumento na transmissão da doença nessa região e no município de Puerto Iguazu-Misiones. Além disso, identificou que as zonas de maior incidência se encontravam em área onde os municípios também fazem fronteira com o Paraguai, o que demonstrou a influência dessa região na transmissão do *Mycobacterium tuberculosis* (4). 

A elevada incidência de tuberculose na parte argentina pode ser devida à alta densidade demográfica, às desigualdades sociais encontradas nos grandes centros urbanos (22) e à zona Norte pertencer à região da tríplice fronteira (4). Esse padrão de adoecimento corrobora o estudo realizado no Nordeste brasileiro que também identificou altas taxas de incidência de tuberculose em grandes cidades e suas regiões metropolitanas (23). 

As regiões de fronteiras internacionais apresentaram maior incidência de tuberculose do que outros recortes espaciais tendo, entre os principais fatores associados a transmissão de doenças: a elevada mobilidade popula­cional, os movimentos migratórios, as atividades de alto impacto ambiental, a falta ou dificuldade de acesso aos serviços de saúde e as condições de vida da população (4,24). Os dados encontrados sobre a incidência de tuberculose neste estudo não permitiram concluir que existe um sobre risco de adoecimento nessa área de fronteira, porém indicaram uma maior influência na transmissão da tuberculose na região da tríplice fronteira.

Na zona leste da área de estudo, onde estão localizados os municípios brasileiros, foram registradas as menores taxas de incidência da doença e identificados alguns municípios sem notificação de casos durante o período total do estudo. Esse dado alerta para a qualidade dos serviços de saúde dessa área com relação ao agravo tuberculose. No Brasil, as maiores incidências de doenças infecciosas e parasitárias, incluindo a tuberculose, são evidenciadas nas regiões Norte e Centro-Oeste e sub-região meio-norte do Nordeste, diminuindo no Sul, onde está Santa Catarina, e no litoral Leste. Essa diminuição está associada, entre outras premissas, a melhores condições de vida da população (25). O índice de desenvolvimento humano municipal dos municípios brasileiros deste estudo variou de médio a muito alto, compatível com boas condições de vida da população (6). 

Essas diferenças destoantes na incidência de tuberculose entre os municípios brasileiros e argentinos indicaram ainda alteração de comportamento do agravo na população podendo ser atribuída a alguns fatores como a influência e condições de vida da população e a qualidade da vigilância, do diagnóstico e do tratamento, pois influenciam no controle da tuberculose (25,26). Pode-se suspeitar também da existência de subnotificação de casos nessas zonas de menor incidência, uma vez que estão localizadas próximas as de elevada transmissão da tuberculose e onde o fluxo de pessoas entre os dois países é elevado. Reconhece-se que, na dinâmica da transmissão do agravo, as interações populacionais são importantes e decisivas na contaminação pelo bacilo da tuberculose (21). 

Na análise dos diferentes períodos de estudo, pré-pandêmico (2009-2019) e período completo (2009-2021), não foram encontradas grandes variações na incidência da doença. Essa área não seguiu a tendência de vários locais pelo mundo que, de maneira geral, relataram diminuição no registro de casos de tuberculose durante a pandemia da covid-19, sendo possíveis causas a subnotificação e a dificuldade de acesso aos serviços de saúde (27). Na Argentina, a taxa de notificação de tuberculose por 100 mil habitantes no período 2014-2019 aumentou, com variação anual média de 3,7%, e, no período 2019-2020, a taxa diminuiu 12,8%, sendo também atribuída, entre outros, à pandemia da covid-19 (17). No Paraná, observou-se diminuição no período pandêmico, diferindo do encontrado neste estudo que não observou essa tendência. A hipótese é de que as notificações de tuberculose nessa área apresentaram tendência crescente mesmo antes do início da pandemia da covid-19 (28).

A análise temporal sugeriu tendências de crescimento na incidência de tuberculose na região de estudo, mas essas tendências não foram significativas. Entretanto, dados demonstraram incremento no Brasil a partir de 2015, onde a incidência de tuberculose era de 34,3 casos por 100 mil habitantes, aumentando para 36,9 em 2018, com estabilização em 2019 (37,1 casos por 100 mil habitantes) (5). Na Argentina, a taxa também aumentou de 22,8 casos por 100 mil habitantes em 2015 para 25,5 em 2021 (17). Essa tendência foi heterogênea nos estados e nas províncias dos dois países e apresentou variações anuais crescentes e decrescentes (5,17). 

Esses incrementos na incidência da tuberculose podem estar relacionados com questões sociais, problemas no diagnóstico, abandono do tratamento, coinfecção tuberculose-HIV (29) e tuberculose drograrresistente. A incorporação de novas tecnologias diagnósticas mais sensíveis no Sistema Único de Saúde pode contribuir para o aumento da incidência (5,30). A Argentina também alcançou avanços no diagnóstico da tuberculose com a introdução de novas tecnologias e treinamentos para os profissionais de saúde (18). Esses dados indicaram que a tuberculose ainda é um problema desafiador para a saúde pública e dificulta o alcance da meta “Estratégia pelo Fim da Tuberculose” da OMS de redução da incidência da doença para menos de 10 casos por 100 mil habitantes até 2035 (30). 

Analisando os municípios de fronteira internacional como área única, foi possível identificar as zonas de maior risco de transmissão da tuberculose, que são prioritárias para as ações de controle da doença, e as de menor incidência, onde se propõe investigar mais detalhadamente o cenário epidemiológico da tuberculose e a atuação da vigilância.

Não foram detectadas mudanças de tendência temporal significativas da incidência de tuberculose, mas foram localizados aglomerados espaciais do tipo alto-alto (município e vizinhos com alta incidência) na Argentina e baixo-baixo (municípios e vizinhos com baixa incidência) no Brasil. Esses resultados sugeriram atuações integradas dos países, como campanhas de diagnóstico em áreas de maior risco, e ações para melhorar a vigilância em áreas de subnotificação. Além disso, apontaram a necessidade de cooperação transnacional para fortalecer o controle da tuberculose em áreas fronteiriças. Isso inclui aprimoramento das políticas públicas, ações bilaterais para financiamento e assistência em saúde, gestão compartilhada de sistemas de informação, bem como serviços de vigilância padronizados.
